# It's all about relationships: A qualitative study of health researchers' perspectives of conducting interdisciplinary health research

**DOI:** 10.1186/1472-6963-8-110

**Published:** 2008-05-23

**Authors:** Kalpana M Nair, Lisa Dolovich, Kevin Brazil, Parminder Raina

**Affiliations:** 1 Centre for Evaluation of Medicines, St. Joseph's Healthcare Hamilton, 105 Main Street East, Hamilton, ON L8N 1G6, Canada; 2 Department of Family Medicine, McMaster University, 75 Frid Street, Hamilton, ON L8P 4M3, Canada; 3 St. Joseph's Health System Research Network, 105 Main Street East, Hamilton, ON L8N 1G6, Canada; 4 Department of Clinical Epidemiology & Biostatistics, McMaster University, DTC, Room 308, 1280 Main Street West, Hamilton, ON L8S 4L8, Canada

## Abstract

**Background:**

Interdisciplinary research has been promoted as an optimal research paradigm in the health sciences, yet little is known about how researchers experience interdisciplinarity in practice. This study sought to determine how interdisciplinary research was conceptualized and operationalized from the researcher's perspective and to better understand how best to facilitate interdisciplinary research success.

**Methods:**

Key informant interviews were conducted with health researchers with expertise or experience in conducting interdisciplinary research. Interviews were completed either in person or over the telephone using a semi-structured interview guide. Data collection occurred simultaneously with data analysis so that emerging themes could be explored in subsequent interviews. A content analysis approach was used.

**Results:**

Nineteen researchers took part in this study. Interdisciplinary research was conceptualized disparately between participants, and there was modest attention towards operationalization of interdisciplinary research. There was one overriding theme, "It's all about relationships", that emerged from the data. Within this theme, there were four related subthemes: 1) Involvement in interdisciplinary research; 2) Why do I do interdisciplinary research?; 3) Managing and fostering interdisciplinary relationships; and 4) The prickly side to interdisciplinary research. Together, these themes suggest that the choice to conduct interdisciplinary research, though often driven by the research question, is highly influenced by interpersonal and relationship-related factors. In addition, researchers preferred to engage in interdisciplinary research with those that they had already established relationships and where their role in the research process was clearly articulated. A focus on relationship building was seen as a strong facilitator of interdisciplinary success.

**Conclusion:**

Many health researchers experienced mixed reactions towards their involvement in interdisciplinary research. A well thought-out rationale for interdisciplinary research, and strategies to utilize the contribution of each researcher involved were seen as facilitators towards maximizing the benefits that could be derived from interdisciplinary research.

## Background

Interdisciplinarity in health research has become a common research paradigm. Globally, central research agencies such as the National Institutes of Health (NIH) in the United States, the Seventh Research Framework Programme (FP7) in the European Union, and organizations such as the World Health Organization (WHO) have all focused efforts towards increasing interdisciplinary research. In Canada, the Canadian Institutes of Health Research (CIHR) has been at the forefront of promoting interdisciplinarity within health research with many funding opportunities geared specifically towards the development of interdisciplinary teams [1]. This shift in how research is funded, along with changes in academia (e.g. joint appointments; interdisciplinary programs and faculties), has further reinforced the value placed by funders and academic institutions on the conduct of interdisciplinary research. Despite this emphasis on interdisciplinarity, relatively little has been documented about how researchers experience interdisciplinary health research in practice.

Although interdisciplinarity is touted as a valuable aspect of health research, it is only recently that there have been attempts to define and operationalize it, with varying definitions in existence. Interdisciplinarity within health sciences research usually involves researchers from multiple disciplines working *together *to tackle complex, multifaceted research questions. In 2005, the Canadian Academy of Health Sciences defined interdisciplinary research as "a team of researchers, solidly grounded in their respective disciplines, that come together around an important and challenging health issue, the research question for which is determined by a shared understanding in an interactive and iterative process" (p.764) [1]. A systematic review in 2007 saw interdisciplinary research explicated as: "any study or group of studies undertaken by scholars from two or more distinct scientific disciplines. The research is based upon a conceptual model that links or integrates theoretical frameworks from those disciplines, uses study design and methodology that is not limited to any one field, and requires the use of perspectives and skills of the involved disciplines throughout multiple phases of the research process" (p.341) [2]. Thus, the presence of at least two disciplines, a shared delineation of the research question, and involvement from each discipline throughout the research process have been noted as key elements of interdisciplinary research. Related types of research, multidisciplinary and transdisciplinary share similar features. Multidisciplinary research typically involves disciplines working more independently on a research study, and transdisciplinary research purports to utilize the methods and perspective of various disciplines to generate new knowledge and approaches. Therefore, each type of research is characterized by different levels of involvement by researchers and has varying impacts on knowledge generation. It is interdisciplinary research, however, that has been the focus of much attention within the last few years regarding its definition.

Scholars such as Julie Thompson Klein [3-5] have been writing about interdisciplinarity since the early 1990s, but it is only in the last five-to-ten years that there has been a marked increase in the nature and frequency of attention devoted to interdisciplinary research. A number of commentaries and articles have described some of the challenges of interdisciplinary research in health and science [1,6-9]. In Canada, government, industry, and academia have both supported and hindered the uptake of interdisciplinary health research [1]. For example, despite many funding initiatives focused on interdisciplinary health research, academic institutions are still primarily organized through disciplinary-based boundaries (and faculty rewarded for contribution to these disciplines), thereby thwarting incentives for moving beyond these boundaries. Others have also cited the tenure system as a major impediment to interdisciplinary research, and as a result some researchers may avoid opportunities for participation in interdisciplinary research [6,8,9]. The push for involvement in interdisciplinary research has left some researchers feeling compelled to undertake interdisciplinary research, concerned about becoming adisciplinary, and feeling frustrated with continually re-educating new disciplines about one's own discipline [8]. As well, within an international research milieu, context is important in shaping research questions and their findings, and there can be difficulties in navigating differences in terminology and culture [10]. These papers have been instrumental in laying the foundation for documenting the nature and realities of interdisciplinary research in health and science. The logical next step is an examination of the experience of researchers currently conducting interdisciplinary health research within academia. Accordingly, this study sought to determine how interdisciplinary research was conceptualized and experienced from the academic health researcher's perspective and to understand how to foster success in interdisciplinary health research. This study was the first phase of a larger study examining evaluation of the interdisciplinary component of health research, and it was felt that researchers' in-vivo experiences and perceptions would be a suitable platform from which to build an evaluation framework for interdisciplinary research.

## Methods

### Design

This was a descriptive study utilizing qualitative interviews to explore the experiences and perceptions of academic health researchers. Although focus groups were a possible data collection method, focus groups would not have allowed for an in-depth examination of individual experiences. The study setting was the university environment as we were interested in understanding the perspective of health researchers working within academia.

### Sampling and data collection

A purposeful approach to sampling was used [11]. Researchers, or key informants, with known experience of or expertise in interdisciplinary health research were invited to participate in this study as we wanted to learn from those who had already engaged in the conduct of interdisciplinary health research. This use of specific inclusion criteria is common when conducting key informant interviews [12]. Often studies that involve key informants start with the investigators drawing up a list of potential participants [12]. In this study, two of the study investigators (LD and KN) generated a small list of possible participants based on their knowledge of the type of research that they did (e.g. researchers completing interdisciplinary health research or having published findings from interdisciplinary research). Researchers who had worked together were not specifically sought however there were participants in this sample who indicated in their interview that they had worked with other participants. Three specific types of purposeful sampling strategies were used in this study, and it is not uncommon for qualitative studies to involve more than one type of sampling strategy [11]. Critical case sampling was utilized to elicit participants who exemplified key characteristics [11]. For example, we explicitly sought to include researchers with backgrounds in statistics and in health policy as it was recognized that these disciplines were inherently interdisciplinary within the health field and that these key informants could therefore provide this unique perspective. Snowball sampling was also used to determine other suitable participants, as we were aware that there were likely key informants that were not known to us at the onset of the study [11]. Finally, maximum variation sampling (seeking a range of participants) was utilized to ensure that data reflected a diversity of experiences. For example, it was evident half way through the interviews that more participants who were earlier in their careers were needed (e.g. junior faculty), and participants reflecting this characteristic were specifically sought.

Potential participants were initially contacted via email by the principal investigator (KN) and provided a copy of the study information sheet and study consent form. Participants were asked to respond to the request for an interview via a return email. If the participants agreed to complete the interview, arrangements were made to either complete the interview in person or over the telephone, depending on the preference of the participant. Those who did not respond to the initial email request were sent another email within a couple of weeks. If after three email attempts, no response was received, no further contact was initiated. All participants were asked to complete one interview.

A semi-structured interview guide was used to explore participants' perceptions and experiences of conducting interdisciplinary health research, potential barriers and facilitators, and knowledge of literature about interdisciplinary research. Detailed questions on evaluation were also asked and will be reported as part of the wider study. All interviews were digitally recorded and transcribed with the participant's consent. Participants were mailed a $25 gift certificate as a token of appreciation after completion of the interview.

Saturation was the main determinant of how many interviews were completed, and data collection stopped when no new information was gained for each of the main themes generated [13]. Personally identifying information was deleted from transcripts during the transcription and data cleaning process, and a coded number identified each participant.

### Data analysis

Data analysis took place concurrently with data collection to ensure that new themes were sufficiently explored. A content analysis approach was used to extract recurrent themes across interviews [14]. All transcripts were coded by the principal investigator [13]. Coding involved reading each transcript and putting like elements of text into broad groupings. Each of these groupings was then read and re-read to establish key themes. Following the delineation of key themes, all interviews were examined for the presence of each theme and for a range of responses within each theme [13]. This coding process allowed for understanding of the breadth and variation in responses that were present in the interviews. A provisional codebook was developed after the first couple of interviews and refined as the analysis progressed. Quotes reflective of emerging themes were extracted as the analysis was conducted and further examined as the paper was written to ensure that these best reflected the interpreted experience of participants. Illustrative quotes are included within findings.

QSR NVivo (version 2), a software program designed for qualitative research, was used to help organize the data.

### Study rigour

This study proceeded once ethics approval has been obtained from the St. Joseph's Healthcare Hamilton Research Ethics Board (#06-2689) in Hamilton, Ontario, Canada. Study rigour was maintained in a number of ways. An audit trail was kept to document reasons for changes to the interview guide, codebook, and themes. All interview transcripts were cleaned prior to coding to ensure accuracy. The principal investigator documented personal perceptions, biases, and beliefs about interdisciplinary research at the onset of the study and periodically examined these as data collection and analysis were taking place in an effort to minimize undue influence of these on the interpretation of the data.

All participants were invited to a presentation of the study findings and given the opportunity to provide feedback about the key themes generated. Any comments provided were incorporated into the final analytic picture. This step (member checking) was completed to ensure that the interpretation of findings reflected participants' experiences and resonated with their perceptions [15].

## Results

Of the 20 people invited to participate, 19 agreed and one declined due to scheduling conflicts. Seventeen interviews were conducted in person, and two were completed over the telephone. Interviews ranged from 17 to 66 minutes. Eleven participants (58%) were female, and 12 (63%) had worked in research for over 15 years. Fourteen participants (74%) had worked on more than 20 studies that they considered interdisciplinary. Twelve participants (63%) were in a leadership position in academia (e.g. Director, Associate Director). A range of disciplines (as identified by participants) were represented in this sample and are noted in Table 1.

**Table 1:** Participant Characteristics

**Self-Identified Primary Discipline (Participant ID #)**	**Leadership position**	**Number of ID studies**	**Number of years in research**
Clinical Pharmacology (1)	Yes	21–25	15
Economics (14)	No	11–15	20
Environmental Health (8)	No	40+	35
Family Medicine (6)	Yes	40+	22
Health Policy (3)	No	6–10	8
Marketing (4)	No	16–20	30
Medicine (specialist) (13)	Yes	40+	25
Medicine (specialist) (19)	Yes	40+	30
Nursing (12)	Yes	26–30	29
Pharmacy (2)	Yes	21–25	9
Philosophy (15)	No	11–15	16
Philosophy (17)	Yes	11–15	27
Political Science (7)	Yes	31–35	9
Social Work (9)	Yes	26–30	15
Sociology (10)	No	26–30	15
Sociology (11)	Yes	40+	20
Sociology (16)	No	6–10	8
Sociology (18)	Yes	40+	36
Statistics (5)	Yes	40+	10

### Conceptualizing interdisciplinary health research

Participants were involved in a variety of interdisciplinary health research studies that included clinical trials, health services research, health policy analysis, environmental health, and patient and clinician-related interventions. However, despite all participants engaging in interdisciplinary research, there were variations in how researchers defined and conceptualized interdisciplinary research. All participants generally agreed that at least two different disciplines were needed for the conduct of interdisciplinary research, although a small minority felt that having a minimum of three or four different disciplines present was a more ideal scenario. Interdisciplinary research was typically conceptualized in very general terms (bringing multiple disciplines together to answer a research question) and there was little use for the distinctions between multidisciplinary, interdisciplinary, and transdisciplinary research. There were two participants, however, who felt that these distinctions were useful and needed. Both of these researchers were among the few interviewed who were more familiar with the literature about interdisciplinary research. Participants typically added investigators with particular skills as a study progressed versus having a complete complement of all disciplines present at the onset of the study. Three participants had conducted and published articles in academic journals about their experiences or perceptions of doing interdisciplinary research.

### Conducting interdisciplinary research

There was one overriding theme that emerged from the data, "It's all about relationships". Research conducted where there was an existing positive relationship was seen as facilitative of knowledge generation and transfer. One of the most commonly cited incentives for doing interdisciplinary research (or continuing to do interdisciplinary research), related to engagement with others where there was a mutual respect, comfort, and in many cases, a past history of working together.

...it [interdisciplinary research] appears not to be thought about consciously, certainly on my part, and I think the more I've done this, the less conscious it becomes, more gets taken for granted in an interdisciplinary context as it would in a disciplinary one. ...I think certainly as you move through and I'm sure you're going to be talking with people at their difference stages of their interdisciplinary careers you'll find that there's people that you can work with well and others that you will never work with again because it wasn't a great experience. [Int8-Environmental Health]

This response was echoed through all of the interviews. It appeared that prior experience with different disciplines working together mitigated the potential for disciplinary division within the research team. One researcher nicely captured this sentiment of balancing the difficulties with the possible benefits that could be derived:

[What is] underappreciated is that relationships develop over time and there's a huge transition cost of establishing one ... I think it has to do with relationships, it has to do with rapport. It has to do too with really creative thinking;, maybe it's too strenuous for me, maybe a smarter person wouldn't have a problem, but I think it is strenuous communicating and getting someone else to understand. Getting a whole room full of people to understand is difficult. But I can say a couple of colleagues with whom I've just hit it off intellectually, we've done, even when there's been a group, we've tended to pull the project along. And I think it's been wonderful and even quite a stretch across totally different methods, totally different backgrounds, so that kind of friendship almost based engagement. I don't know which comes first, the collaboration I guess and then friendship in most cases. [Int10-Sociology]

Researchers valued the ability to build upon existing relationships and some felt that this focus contributed most towards new knowledge development. Working in large teams (more than five or six people) was not seen as a productive mode of research; often working closely with one to three people was seen as the ideal as a smaller group would better allow for focused attention and integration regarding what each discipline could contribute to the study.

Within this primary theme, there were four related subthemes: 1) Involvement in interdisciplinary health research; 2) Why do I do interdisciplinary research?; 3) Managing and fostering interdisciplinary relationships; and 4) The prickly side to interdisciplinary research. Taken together, they offer a picture of how interdisciplinary research was experienced by the interviewees as a group, why some researchers chose to involve themselves in interdisciplinary health research, and how some of the challenges they experienced were managed.

#### 1. Involvement in interdisciplinary health research

For many, forming an interdisciplinary team of researchers was not a conscious decision but was inherent to the type of research questions they studied. Those with interdisciplinary backgrounds also tended to find themselves working predominantly with interdisciplinary teams:

I think that because I don't easily fall into one particular discipline, I've always just been at the interface of a bunch of different domains, and certainly the teams I work in are typically like that. ... I do think that our work is so fundamentally at the juncture of so many domains, it's just how we do it [research] and I think it's [interdisciplinary research] just second nature to us individually and as groups. [Int7-Political Science]

Importantly, a number of participants commented on the necessity of recognizing that not all research questions require an interdisciplinary approach, with many questions being appropriate for single disciplines to investigate:

And not all questions need to have a multidisciplinary or interdisciplinary team working on a project. Some questions don't need that; some questions can be very well answered within disciplines. We can't loose sight of that either. [Int2-Pharmacy]

There were also researchers who discussed the appropriateness of when and who should engage in interdisciplinary research. There was a widespread recognition that interdisciplinary research could be disadvantageous to more junior researchers, and that some seniority was needed in order to effectively negotiate the complexities of interdisciplinary work:

When you go into interdisciplinary work ... you have to be very good at boundary setting...and you have to be prepared to do the political balancing act to what you say yes to and what you don't. I don't think you can do it as a junior scholar. I really believe that you do that at your peril. You have to have some seniority and some political clout to move into it [interdisciplinary research]. [Int9-Social Work]

Many senior investigators commented that interdisciplinary research was not valued equally when tenure and promotion was being considered and that junior faculty may not be in a position to negotiate otherwise. A revisiting of tenure and promotion criteria in some departments was felt necessary to truly foster an academic milieu of open interdisciplinary research. For example, one researcher described a scenario whereby a colleague opted not to join an interdisciplinary research team, as his department did not recognize the contribution of multi-authored papers in the tenure and promotion process.

#### 2. Why do I do interdisciplinary research?

Participants were asked to talk about what motivates them to conduct or continue to be involved in interdisciplinary research. There were four factors consistently noted by participants as contributing towards this pursuit: 1) the nature of the research question, 2) opportunities for funding, 3) opportunity to learn about something new/see problem in a new light, and 4) the ability to have a stronger impact on knowledge transfer and uptake.

The central driver for engaging in interdisciplinary research was the need for different knowledge sectors to contribute towards understanding a complex health research area; this sample was predominantly comprised of a seasoned group of researchers who recognized that many health questions could not be answered effectively within a single discipline.

On a more pragmatic note, most researchers took advantage of funding opportunities that arose within an environment of shrinking research dollars; being opportunistic was a necessity for survival within academia. Experienced researchers expressed frustration at the almost forced interdisciplinarity that funding agencies imposed, and how this focus often led to inefficient and less productive research. Some reflected that multidisciplinary research would have been more effective as this type of research would have allowed each discipline to bring their expertise to the study in a more focused manner. Ultimately, having a strong, well-defined rationale for interdisciplinary research was seen as a key facilitator towards research success.

The opportunity for exposure to new methods or theories was cited by some participants. In most cases, this experience was simply an antecedent bonus to doing interdisciplinary work, as it was generally not practical to develop a research proposal solely around an area that one was interested in learning more about.

Finally, for many researchers there was also a strong belief that different knowledge bases would be able to contribute towards the creation of stronger solutions and answers, thus leading to more meaningful and useful results. Interdisciplinary research was seen as something that could foster uptake of research findings:

... and the [research] product was different due to team composition, and the product was better and by the time that research was done... that project went forward to the government and has been implemented, where it could have just sat being critiqued... [Int12-Nursing]

#### 3. Managing and fostering interdisciplinary relationships

The importance of cultivating interdisciplinary relationships was raised by many participants, and leadership and role clarification were cited as drivers that could foster this development. Without a strong leader to guide the interdisciplinary research process, the potential contributions of researchers from other disciplines could not be effectively realized.

But it also takes a lot of leadership to make that [interdisciplinarity] possible. You could have an interdisciplinary group that is completely dysfunctional because there's nobody to actually give it a sense of direction. Anytime you have a group, I mean even a group of well-qualified individuals, they often sometimes need direction. So having someone who is good in directing people or at least providing some sense of direction really helps. [Int5-Statistics]

Involvement in a team that fostered the input of all team members was an expectation of most participants and this focus was seen as the responsibility of the principal investigator.

The role that each researcher assumed varied based on how the team was initially constructed. Involving all researchers in most aspects of the study was seen as a way to keep team members engaged and participating. For example, one researcher described a study where some of the team members had never been involved with empirical research and the principal investigator had all researchers take part in the data analysis:

I actually insisted on [everyone taking part in data collection & analysis]. Because I thought in order to have a meaningful discussion about the material...everyone should be involved in at least two interviews and one level of analysis of part of the dataset. [Int3-Health Policy]

Other participants discussed the importance of clarifying the role of each team member. This elucidation was seen as critical for research success as it helped to ensure that everyone on the team was aware of what each other was contributing to the team.

I think one of the things is to fairly early on have a discussion about what each team member brings and even more importantly why they were asked to join the team. [Int12-Nursing]

Participants also articulated how interdisciplinary research itself influenced the researcher-team relationship and how one participated in the team. One participant noted that interdisciplinary research involved a high level of confidence to be able to acknowledge gaps in one's knowledge:

One has to be reasonably comfortable with oneself and to the point where you say," I don't know anything about this. I'm a learner in this part of this study and I can't even challenge, apart from being a naive listener saying, well, from my perspective I hear this". So it takes a lot of maturity and self-confidence when you're doing that kind of work to even enter into it because it [interdisciplinary research] is going to take longer, it is going to cause you to be uncomfortable frequently with what you don't know. [Int3-Health Policy]

#### 4. The prickly side to interdisciplinary research

There were mixed feelings expressed about involvement in interdisciplinary research. Most barriers related to the large time investment needed to effectively coordinate and work with a team of disparate disciplines. A few people noted the challenges of working across distances when doing interdisciplinary research and it was typically recommended to work with people where face-to-face meeting was possible.

A general theme that proliferated the interviews was the notion that interdisciplinary research simply involved an understanding of interpersonal dynamics and how to deal with differing personalities, irrespective of the discipline that they come from. In many interviews, researchers highlighted how challenging personal dynamics could be:

And the other thing is the personalities, anytime you're dealing with different people, there's always issues of differences in culture. And our educational backgrounds are cultures, so personalities also sometimes make it difficult in an interdisciplinary environment. [Int5-Statistics]

Taking time to build interdisciplinary relationships was cited as a key factor that could enhance the conduct of interdisciplinary research. Time to build relationships was seen as both a necessity but also as a frustration as sometimes the short time frame of grants did not allow for this development. Time was needed to learn about the perspective of others, assess the value of what they are contributing, and finally, to assimilate this new knowledge into one's own knowledge base. For some, the length of the relationship-building process obscured the possibility of more informed solutions and learning that could be gained from interdisciplinary work. Overall, participants were attuned to the practicalities of conducting interdisciplinary health research, versus taking time to reflect on their experience of it.

Marginalization within an interdisciplinary team was also an ongoing concern, and this typically resulted from roles and expectations not being clearly delineated. Some people recognized that power imbalances could exist and made a conscious effort to articulate when this occurred, while others felt limited in their ability to openly address this marginalization. Dealing with these dynamics was seen as a disincentive towards being involved with interdisciplinary teams.

The other part of interdisciplinarity that doesn't feel talked about much is power...sometimes it feels like we have just one discipline [present] with all of these smaller voices on the edge. [Int16-Sociology]

### Interdisciplinary Success

Participants in this study offered possible solutions to three key challenges of interdisciplinary health research that were consistently raised during the interviews: 1) not understanding what interdisciplinarity research is really about; 2) varying personalities and viewpoints; and 3) marginality and power dynamics. Ideas for maximizing the potential benefits of interdisciplinary research emerged from the interview discussions and are summarized in Table 2. In general, an explicitness about the role and contribution of each discipline was seen as critical for facilitating a smooth research process. Strong leadership by the principal investigator throughout the research study could also keep all disciplines engaged and could minimize the power differentials between the various disciplines involved.

**Table 2:** Maximizing the benefits of interdisciplinary health research

**Challenge**	**Description**	**Possible solutions for addressing challenge**
Not understanding what interdisciplinary research is really about	▪Researchers come to the table without fully appreciating the intent of interdisciplinary research study	▪The research team together establishes how they define and view interdisciplinarity for their study
		▪A plan to assess whether the research team is meeting their goals regarding interdisciplinarity is in place
		▪Regular assessment/refinement of progress is conducted
Varying personalities and viewpoints	▪The focus of the research study is derailed by those with their own agenda/perspective	▪Active leadership by principal investigator to keep all disciplines engaged and contributing
		▪Regular interaction/communication by team members
Marginality and power dynamics	▪Some research team members minimize and exclude the contribution of other disciplines in the research team	▪Explicitness re: each person's role & contribution to the study is established at the onset of the study
		▪Regular assessment of each discipline's contribution is built into the study plan

## Discussion

This study examined how interdisciplinary research was conceptualized and experienced by researchers involved in health research. Researchers valued interdisciplinary research as a mechanism for more completely answering complex health questions and they appreciated the potential advances in how knowledge was generated and the possible impacts of this new knowledge. However, many researchers described mixed reactions towards participation in interdisciplinary research. The challenges of managing different personalities, working with large numbers of people, and the time needed for effective relationship building were seen as disincentives to interdisciplinary research. Nevertheless, a well thought-out rationale for interdisciplinary research, strong leadership, an attention to power imbalances, and strategies in place to maximize the contribution of each researcher involved were seen as facilitators towards taking advantage of the benefits that could be derived from interdisciplinary research. Importantly, researchers consistently talked about the critical role of relationships in fostering interdisciplinary success.

Participants in this study cited many benefits (opportunity for greater impact; new knowledge generated; learning new methods) and challenges (managing different personalities; tenure criteria; time investment) of interdisciplinary research that have been previously described in the literature [8,9,16-19]. Of particular note was the recurrent sentiment that interdisciplinary research could be detrimental to the careers of junior faculty due to the lack of recognition that it has in some tenure and promotion criteria. This concordance with previous findings helps to consolidate knowledge about the main drivers for academic health researchers towards engagement in interdisciplinary research. Participants generally agreed on the value of conducting interdisciplinary research. However, most researchers conceded that new disciplines are often brought into teams when particular skills are needed, and not necessarily at the proposal development stage. These researchers did not view this deviation as indication that their research was perhaps more accurately multidisciplinary [20] in nature; they felt that engagement with new disciplines at specific junctures in a study could still lead to new learnings and directions in the research.

Interdisciplinary research was unconsciously undertaken by some participants and was seen as intrinsically part of their research scope. Interdisciplinary research conduct as "second nature" has also been described by those who have undertaken interdisciplinary graduate programs [21]. These researchers highlight their ability to bridge the divide between disciplines given their knowledge and exposure to a variety of methods and approaches [21]. It may be that there is an emerging breed of researchers who may be better equipped to manage the challenges of interdisciplinarity in health research due to their early exposure of working in an interdisciplinary manner.

Much of the scholarly writing about interdisciplinarity is situated within a discourse of how knowledge is constructed [3,5,20,22,23]. The researchers interviewed focused primarily on the practicalities of conducting interdisciplinary research (e.g., managing group dynamics). In addition, all researchers were asked to share information about literature regarding interdisciplinarity and interdisciplinary research, and few were aware of the vast compilation of literature related to interdisciplinarity. This gap in knowledge highlights the focus of this sample on 'getting the research done" with few having time to deeply ponder epistemological debates. This lack of explicit attention towards epistemology and knowledge construction may also be reflective of this sample's focus on one type of research, health research, which may have more fluid disciplinary boundaries and foundations.

These findings are important as they offer insight into the motivations of health researchers towards conducting interdisciplinary research. Although all participants were health researchers, there were 15 distinct disciplines represented within the sample. The general uniformity of experiences and perceptions across these disciplines implies a universality of factors that could be delineated for successful interdisciplinary research. For example, many researchers found that they favored working with those with whom they had pre-established relationships. This preference suggests that when forming an interdisciplinary health research team, starting with a core group of researchers who have already worked together and then adding a minimum of new researchers could help to ameliorate typical interdisciplinary "growing pains". Strong leadership and clearly delineating each person's role on the research team can also help to minimize difficult group dynamics.

This study is not without its limitations. Some participants had difficulty separating their experiences within interdisciplinary research teams from non-interdisciplinary research teams. Despite efforts to re-orient participants back to an interdisciplinary focus, it is possible that some comments will have been more general in nature than expected. The study findings emphasize the challenges of disentangling general group dynamics from interdisciplinary group dynamics. Also, some interviews were shorter than expected and in one case it was evident that the interview was 'fit in' between other commitments. Although the findings generated from these shorter interviews were useful, the difficulties of engaging busy health academics (and in some cases clinicians) in a qualitative study were affirmed. This study sample was also comprised of researchers more seasoned in the conduct of interdisciplinary health research, despite efforts to recruit junior investigators. As a result, the transferability of findings may be limited. Furthermore, this study's exclusive focus on the perspective of researchers involved in interdisciplinary health research may also impact the transferability of findings to other types of researchers and those working outside of academia. Future research examining the views and experiences of researchers from a broad spectrum of environments and foci would be of interest. Finally, few participants engaged in the member checking exercise and greater participation would have provided stronger assurance that the findings accurately reflected their perceptions.

## Conclusion

The current pressure to be involved in interdisciplinary research has created a scenario where interdisciplinarity is viewed with mixed emotions. Dalke et al [23] remind us that "interdisciplinarity is not a place to be reached" but evolves as process of working where one is open towards seeing the world in different ways. They see interdisciplinarity as a "freeing" process that liberates one from feeling that one "must know it all" [23]. Unfortunately, the pressured funding climate and sometimes one-dimensional approach towards promotion in academia, has created an atmosphere that has made involvement in interdisciplinary research less desirable, particularly early in one's career. By documenting actual struggles and possible remedies, this study provides a place from which the development of an organizing framework for the successful conduct of interdisciplinary health research is possible. A focus on relationship building is one path that can facilitate a positive interdisciplinary experience.

## Competing interests

The authors declare that they have no competing interests.

## Authors' contributions

KMN and LD conceived of the study. KMN collected the data. All authors provided input into the data collection and analysis stages of the study. KMN drafted the manuscript and all authors read and approved the final manuscript.

## Pre-publication history

The pre-publication history for this paper can be accessed here:


